# Investigation of Acute Flaccid Paralysis Reported with La Crosse Virus Infection, Ohio, USA, 2008–2014

**DOI:** 10.3201/eid2312.170944

**Published:** 2017-12

**Authors:** Morgan J. Hennessey, Daniel M. Pastula, Kimberly Machesky, Marc Fischer, Nicole P. Lindsey, Mary DiOrio, J. Erin Staples, Sietske de Fijter

**Affiliations:** Centers for Disease Control and Prevention, Atlanta, Georgia, USA (M.J. Hennessey, D.M. Pastula);; Centers for Disease Control and Prevention, Fort Collins, Colorado, USA (M.J. Hennessey, D.M. Pastula, M. Fischer, N.P. Lindsey, J.E. Staples);; Ohio Department of Health, Columbus, Ohio, USA (K. Machesky, M. DiOrio, S. de Fijter)

**Keywords:** arbovirus, *Bunyaviridae*, *Orthobunyavirus*, La Crosse virus, acute flaccid paralysis, paralysis, weakness, neurologic, Ohio, viruses, United States

## Abstract

Infection with La Crosse virus can cause meningoencephalitis, but it is not known to cause acute flaccid paralysis (AFP). During 2008–2014, nine confirmed or probable La Crosse virus disease cases with possible AFP were reported in Ohio, USA. After an epidemiologic and clinical investigation, we determined no patients truly had AFP.

La Crosse virus is an *Orthobunyavirus* transmitted to humans primarily through the bite of *Aedes triseriatus* (eastern treehole) mosquitoes; the virus is endemic to the midwestern and Appalachian areas of the United States (*1**,**2*). Infection with La Crosse virus can result in severe disease, such as meningoencephalitis, which is primarily seen in children. Most infections do not usually result in permanent motor sequelae, but some have resulted in longer term learning difficulties and behavior changes (*2*). Although other mosquitoborne viral diseases (e.g., West Nile virus) have been associated with acute flaccid paralysis (AFP)—a clinical syndrome of rapidly progressive flaccid paralysis typically caused by dysfunction of the anterior horn cells, peripheral motor nerves or nerve roots, neuromuscular junction, or muscle—La Crosse virus has not been identified as a cause (*3**,**4*). During 2008–2014, nine of the total 148 confirmed or probable La Crosse virus disease cases in Ohio were reported as having AFP by local health departments, per the national arboviral case definition (*5**–**7*). We performed an investigation to describe the clinical features and outcomes for these cases and determine if the La Crosse virus infections were associated with AFP.

## The Study

We reviewed medical records from all 9 case-patients’ initial illnesses and abstracted data using a standard form. We contacted patients and, after obtaining consent, administered a questionnaire to assess any residual weakness in the patients and their current functional status according to the modified Rankin scale (a standardized measure of disability for persons with neurologic conditions, by which a score of 0 is normal and 5 denotes severe disability) (*8*). If the patient was <18 years of age at the time of the follow-up interview, we obtained permission from a parent or guardian and administered the questionnaire to the parents. For patients with unclear neurologic findings or diagnosis, a board-certified neurologist performed a follow-up neurologic examination. We obtained human ethics approval for this investigation from the Centers for Disease Control and Prevention Institutional Review Board.

We defined AFP as a clinical syndrome of rapid-onset extremity weakness, sometimes accompanied by weakness in a facial or respiratory muscle that is flaccid (i.e., without any upper motor neuron signs in the affected areas suggestive of central nervous system motor tract pathology) (*4*). Upper motor neuron signs included increased muscle tone or spasticity, increased deep-tendon reflexes, the presence of Babinski and Hoffmann signs, abnormally slow finger or toe taps out of proportion to weakness, or a clear upper motor neuron pattern of weakness (e.g., hemiparesis involving the face, arm, and leg). In addition, a brain lesion that corresponded to the patient’s weakness identified by magnetic resonance imaging (MRI) or suggested by electroencephalography (EEG) provided corroborating evidence of an upper motor neuron or central nervous system motor tract pathology that would not be consistent with AFP. Finally, any residual weakness with a clear upper motor neuron pattern (e.g., hemiparesis involving the face, arm, and leg) or any residual upper motor neuron signs in the previously affected areas were also used to rule out AFP.

Of the 9 La Crosse virus disease case-patients with AFP, 8 had confirmed infections and 1 probable. At the time of disease diagnosis, the median age of the 9 case-patients was 12 years (range 4–78 years); 5 (56%) were male. Illness onset dates ranged from September 2008 to September 2013, and case-patients were residents of 8 counties located throughout the state. During their acute illness, all patients experienced fever, altered mental status, and weakness (Table 1). Other common symptoms that patients reported were seizures (7, 78%), headache (7, 78%), and nausea or vomiting (6, 67%). After a median hospitalization of 17 days (range 5–28 days), 7 patients were discharged to their homes, 1 patient to an acute rehabilitation facility, and 1 to a skilled nursing facility.

**Table 1:** Clinical symptoms reported at time of acute illness for 9 patients with La Crosse virus infection and reported acute flaccid paralysis, Ohio, USA, 2008–2014

Clinical sign/symptom	No. (%) patients
Fever	9 (100)
Altered mental status	9 (100)
Weakness	9 (100)
Seizure	7 (78)
Headache	7 (78)
Nausea/vomiting	6 (67)
Muscle pain/myalgia	1 (11)

Of the 9 case-patients, we reached 7 (78%) by telephone to determine their current clinical status; 1 patient could not be reached for follow-up, and the other patient had died (Table 2). The cause of death for this patient was unclear, but after we reviewed the medical and death records, we did not believe it was directly associated with La Crosse virus infection. Of the 7 patients we evaluated, 2 (29%) reported residual focal weakness related to their previous La Crosse virus disease, and 3 (47%) had a current modified Rankin scale score of >1 (median 1), indicating some sort of residual disability.

**Table 2:** Clinical characteristics and follow-up for 9 patients with La Crosse virus infection and reported acute flaccid paralysis, Ohio, USA, 2008–2014*

Case-patient no.	At time of acute illness							At time of follow-up investigation			
	Age, y/sex	Upper motor neuron†			Brain lesions‡			Upper motor neuron†		Reported weakness	Modified Rankin score§
		Signs	Pattern		MRI	EEG		Signs	Pattern		
1	12/F	Yes	Yes		Yes	No		–¶	–	No	0
2	4/M	Yes	Yes		Yes	No		–	–	–	–
3	12/M	Yes	No		Yes	Yes		–	–	No	0
4	12/M	Yes	No		Yes	No		–	–	–	6#
5	78/F	Yes	No		Yes	–		–	–	Yes	3
6	8/M	No	Yes		Yes	–		–	–	No	0
7	13/M	No	Yes		–	Yes		–	–	No	0
8	7/F	No	No		–	–		Yes	No	No	1
9	12/F	No	No		–	–		Yes	Yes	Yes	1

*EEG, electroencephalography; MRI, magnetic resonance imaging. †Upper motor neuron signs included increased tone, increased reflexes, Babinski sign, or abnormally slow finger or foot taps, and patterns of weakness included hemiparesis or weakness preferentially involving the distal extensor muscles. ‡A brain lesion identified by MRI or EEG consistent with the patient’s pattern of weakness provided corroborating evidence of an upper motor neuron pathology. §Modified Rankin score measures disability or dependence in daily activities for patients who have had a stroke or other neurologic disability. Scores range 0–6 as follows: 0, No symptoms; 1, No substantial disability and carries out all usual activities despite some symptoms; 2, Slight disability and able to look after own affairs without assistance but unable to carry out all previous activities; 3, Moderate disability requiring some help but able to walk unassisted; 4, Moderate disability requiring help with bodily needs and unable to walk unassisted; 5, Severe disability that requires constant nursing care; 6, Deceased. ¶Information was not available or not assessed. #Patient had a primary immunodeficiency that required chronic steroid replacement. Several years after his acute La Crosse virus disease, the patient experienced acute gastroenteritis and subsequently died of an unknown etiology; no autopsy was performed.

We saw in our review of case-patients’ medical records that all 9 had rapid-onset focal muscle weakness during their illness. Seven patients had upper motor signs or a clear upper motor neuron pattern of weakness in the affected areas and were therefore determined not to have AFP. Furthermore, all 7 had evidence of a brain lesion that corresponded to their weakness pattern (5 by MRI, 1 by EEG, and 1 by both MRI and EEG). For the other 2 patients, we found insufficient evidence in the medical records to determine whether they had AFP and performed in-person neurologic exams. One patient had no residual weakness but had residual upper motor neuron signs in the previously affected areas. The other patient had both residual weakness and upper motor neuron signs in the affected areas. Therefore, none of the 9 cases were determined to have AFP by our definition.

## Conclusions

Although all 9 La Crosse virus disease case-patients we investigated had acute weakness or paralysis, none met the definition for AFP. The most likely explanation for the initial AFP reports was difficulty in distinguishing flaccid or lower motor neuron weakness due to peripheral nervous system pathology (i.e., pathology at or distal to the anterior horn cells) from spastic or upper motor neuron weakness due to central nervous system pathology (i.e., pathology in the spinal cord or brain motor tracts). Thus, we found no evidence of AFP being associated with La Crosse virus infection. 

To further assist in classifying cases as having AFP, we propose the following surveillance definition for AFP, based on a World Health Organization technical document: a clinical syndrome of rapid-onset extremity weakness, sometimes accompanied by facial or respiratory muscle weakness, that is flaccid (i.e., without any upper motor neuron signs in the affected areas suggestive of central nervous system motor tract pathology) (*4*). AFP can include clinical syndromes caused by acute dysfunction of the anterior horn cells (e.g., acute flaccid myelitis), peripheral motor nerves or nerve roots (e.g., Guillain-Barré syndrome), neuromuscular junction (e.g., botulism or myasthenic crisis), or muscle (e.g., acute myopathies) (*3**,**4*). Rarely, a sudden-onset spinal cord injury or myelopathy can cause a temporary AFP referred to as spinal shock, although it is typical for upper motor neuron signs to develop eventually in these cases (*4*). The Centers for Disease Control and Prevention will continue to work with public health partners to adopt a standardized surveillance definition for AFP, which we hope will mitigate the risk of an incorrect association between certain infections, like La Crosse virus, and AFP.
